# Optimization of continuous EEG monitoring in the inpatient setting- a quality improvement study

**DOI:** 10.3389/fneur.2025.1643069

**Published:** 2025-12-03

**Authors:** Vijaya Lakshmi Valaparla, Tripti Sharma, Mohammad Almomani, Muhammad Hafeez, Fnu Komal, Todd Masel, Diosely C. Silveira, Xiangping Li

**Affiliations:** Department of Neurology, University of Texas Medical Branch, Galveston, TX, United States

**Keywords:** 2HELPS2B score, inpatient EEG monitoring, critical care EEG monitoring, continuous EEG, long term EEG

## Abstract

**Background:**

Continuous EEG (cEEG) is widely used in the inpatient setting to detect non convulsive seizures and status epilepticus. Prolonged EEG monitoring can increase healthcare cost burden and patient discomfort. Optimizing cEEG by safely reducing the duration of EEG monitoring can be done using a validated scoring system. 2HELPS2B score predicts seizure risk in the next 24 h based on first hour of EEG monitoring and provides recommendation on the total duration of EEG monitoring. We aimed to safely reduce the duration of cEEG monitoring in low-risk patients (2HELPS2B score </=2) and optimize resource utilization.

**Methods:**

This study was performed as a quality improvement interventional study from January till April 2025 in the inpatient setting across three campuses of the University of Texas Medical Branch at Galveston, Texas. A 2-step smart phrase was created on the Epic electronic medical record (EMR) for 2HELPS2B score. This was made live on Epic on January 1, 2025. Epileptologists were educated through departmental grand rounds and timely reminders to incorporate the score by including the smart phrase in the first segment of the cEEG reports.

**Results and conclusion:**

The mean monthly duration of cEEG in low-risk group patients reduced by 22.5% in post intervention group. None of the patients in the low-risk group had seizures. Effective implementation of 2HELPS2B scoring system in clinical practice can safely reduce the duration of EEG monitoring in the low-risk group. This will optimize EEG resource utilization and reduce healthcare costs.

## Introduction

Continuous video electroencephalography (cEEG) monitoring is a highly valuable tool utilized in the inpatient setting to detect non convulsive seizures and status epilepticus. This tool guides physicians and intensivists in timely interventions and treatment adjustments that reduce morbidity and mortality. In the past decade the use of cEEG has increased tremendously and is used not only for seizure detection but also for targeted interventions by detecting neurological complications such as vasospasm in subarachnoid hemorrhage and neuro-prognostication (1). Evidence also suggests that cEEG improves patient outcomes by reduction in mortality and morbidity in the critical care setting (2).

Despite being a valuable diagnostic tool, there are several practical implications that may impact its clinical utility. EEG can be laborious and costly, especially when done during after-hours (3). Prolonged duration of EEG monitoring can cause patient discomfort and is prone to artifacts necessitating frequent lead adjustments. This can add to the increased workload of the EEG technologists, increased hardware utilization and resource consumption. In resource limited settings, allocating prolonged continuous EEG monitoring to some patients may restrict access for others. Nonetheless, the use of long-term EEG monitoring in the United States continues to expand. For example, long-term video EEG Medicare claims increased more than 100 percent in a five-year period, from 53,000 in 2009 to 115,000 in 2014 (4).

Optimizing the duration of EEG monitoring can help mitigate the high-cost burden and reduce the workload of the EEG technologists as well as epileptologists who read the EEGs. This should be ensured alongside providing optimum and highest quality care to the patients that require EEG monitoring.

A prior retrospective observational study showed that patients on cEEG with no epileptiform discharges within the first 4 h were less likely to develop any seizures over the following 18 h (5). Subsequent studies further supported this observation, showing that the longer an EEG remainedfree of epileptiform activity, the lower the probability of subsequent seizures (6). Prediction of seizure risk in a patient can be done by a combination of clinical and EEG criteria (7). These findings led to the idea of using the early EEG abnormalities to triage the duration of EEG monitoring. The presence of certain EEG abnormalities like Lateralized Periodic Discharges (LPDs), Lateralized Rhythmic Delta Activity (LRDA) and Generalized Periodic Discharges (GPDs) were associated with increased risk of seizures (8). The 2HELPS2B scoring system is a validated tool designed to predict 72-h seizure risk based on EEG patterns observed within the first hour of monitoring (9). This scoring system assigns points using certain EEG features and history of any prior seizure (obtained from the patient’s history), with higher scores indicating greater seizure risk. These EEG features include (a) presence of LPDs, LRDA or Bilateral independent periodic discharges (BIPDs) (b) presence of plus features (superimposed fast, sharp or rhythmic activity) with LPDs, LRDA or BIPDs; (c) any rhythmic or periodic discharge except generalized rhythmic delta activity (GRDA)with a frequency greater than 2 Hertz; (d) sporadic epileptiform discharges, (e) presence of Brief potentially ictal rhythmic discharges (BIRDS). Each component is scored with one point except BIRDS which scores 2 points. For each score the risk of seizure in the next 72 h is assessed and a specific total duration of EEG monitoring is recommended. A score of 2 or less is considered low risk. The recommended duration is 1 h for a score of 0, 12 h for a score of 1 and 24 h for a score of 2. For any score more than 2, recommended total duration is at least 24 h (7) (Table 1). This tool has been validated for predicting seizure risk and recommending the total duration of EEG monitoring based on the findings in the first 1 h of EEG monitoring (10). This tool has also been validated for seizure risk assessment in specific neurocritical settings like patients with traumatic brain injury (11). In such situations risk stratification could help guide the recommendations on the total duration of EEG monitoring.

**Table 1:** (A,B) 2HELPS2B score, risk of seizures and recommended EEG duration per Struck et al. (10).

(A)	
Risk factor	Points
Frequency >2 Hz	1
Sporadic epileptiform discharges	1
LPD/BIPD/LRDA	1
Plus features with LPDs/BIPDs/LRDA	1
Prior seizures	1
BiRDS	2
Total score	

(B)							
Total score	0	1	2	3	4	5	6
Seizure risk	<5%	12%	27%	50%	73%	88%	>95%
Recommended total duration of EEG monitoring in hours	1	12	24	>24	>24	>24	>24

## Aim

Our study was performed as a quality improvement initiative to utilize the 2HELPS2B scoring system in the inpatient long-term EEG reports consistently with a recommendation of total duration of EEG monitoring based on the first segment of the report. We aimed at safely reducing the total duration of EEG monitoring for low-risk group (score of =/<2) by at least 30% as compared to the pre-intervention period.

## Methods and metrics

**Figure 1:**
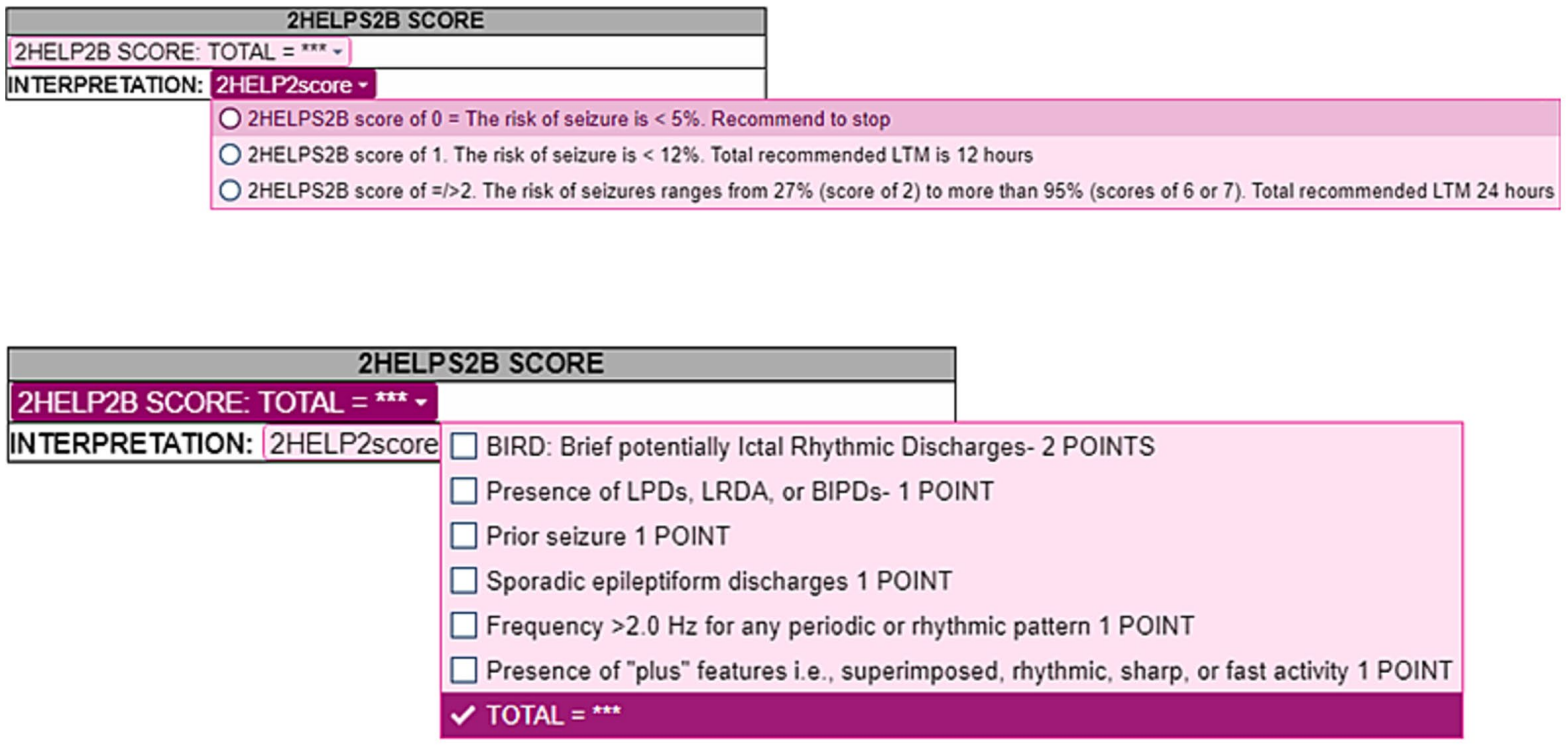
2- Step Epic smartphrase for 2HELPS2B scoring.

This study was conducted at The University of Texas Medical Branch across its three campuses from November 2024 till April 2025. Approval from the Institutional Review Board was obtained. Electronic Medical Record (EMR) was reviewed for all the Longterm EEG orders from November 2024 till April 2025. An epic smart phrase for 2HELPS2B score was created on EMR. This smart phrase is made live on January 1, 2025, for all the epileptologists to incorporate it in the inpatient long term EEG reports (Figure 1). This smart phrase was included at the end of the EEG report for the segment adds the total 2HELPS2B score, the percentage risk of seizures in next 24 h and the recommended total duration of the EEG monitoring. This timepoint when the smart phrase was made live on the EMR was marked as the intervention point. Two months prior to the intervention point, i.e., November and December 2025, was considered pre implementation phase. Post implementation phase started from the day 2HELPS2B smart phrase went live on EMR, i.e., January 2025 till April 2025. Patients above 18 years of age were included in the study. Pediatric patients were excluded from the study. Timely reminders were sent to the epileptologists to improve and consistently utilize the smart phrase in the long-term EEG reports. Patients who were included in the study group had a 2HELPS2B score of </=2, which falls under low-risk group. Patients who had seizures while on EEG monitoring were excluded from the comparison analysis as the study focused on reduction in the mean duration of cEEG in low-risk patients.

EEG reports were reviewed. Pre and post intervention data on the duration of EEG for individual patients was obtained from the EMR. The average duration of the EEG monitoring per month was obtained. Data on demographic profile including age, gender, history of prior seizure and the primary indication of EEG monitoring were obtained from the EMR chart review. Clinical parameters like primary indication for EEG and patient’s primary service team were obtained from the chart review. Age was described as mean and range. Gender, EEG indication and primary service team were described as percentage frequency.

## Results

There were 55 patients in the preintervention group. Fifty-one met the inclusion criteria. Mean duration of the EEG monitoring in November (*n* = 29) was 26.25 h and in December (*n* = 22) was 28 h. 2HELPS2B score was calculated retrospectively. Four patients who had seizures were excluded from the comparative analysis; these patients had a mean 2HELPS2B score of 5, with all scoring >3, thus falling in the high-risk group. Of the 51 patients who did not have seizures, 6 patients had a 2HELPS2B score of 3 and 45 patients had a score of ≤2. Mean duration of EEG monitoring in patients with a score of 0 (*n* = 14) was 22.4 h, patients with a score of 1 (*n* = 22) was 27.6 and patients with a score of 2 (*n* = 9) was 30.8 h. This was as opposed to the recommended duration of 1, 12, and 24 h in groups with scores of 0,1 and 2, respectively.

There were 120 patients in the post intervention group. Nineteen patients were excluded based on the exclusion criteria of having seizures during the EEG monitoring. The mean age of the patients meeting the inclusion criteria (study sample, *n* = 101) was 55.8 with the age group ranging from 20 to 85 years old. Thirty-nine patients were female. Primary indication for EEG was Seizure/ Epilepsy in 22.8%, Altered Mental Status (AMS) in 27.7%, Intracranial bleed/ Brain mass in 12.9%, Cardiac arrest in 6.9% and Others in 29.7%. Most of the sample patients were from Internal Medicine (40.7%) as their primary service, while 19.8% were from Neurology service. Neurocritical Care Unit (NCCU) was the primary service for 5.9% of the patients while Medical Intensive Care Unit (MICU) had 3.9%. The rest of the patients were from other services. Most of the patients were discharged to home (46.5%) or a nursing facility (51.5%) and mortality was observed in 1.2% of the patients (Table 2).

**Table 2:** Demographic and clinical profile of the sample patients.

Variable	Sample size (*N* = 101)
Mean age/range	55.8 years/ 20–85
Gender (female)	36/101
Primary indication for EEG	Seizures/epilepsy (22.8%) Altered mental status (27.7%) Intracranial bleed/ brain mass (12.9%) Cardiac arrest (6.9%) Others (29.7%)
Primary service	Internal medicine (40.7%) Neurology (19.8%) Neurocritical care unit (5.9%) Medical intensive care unit (3.9%) Others (29.7%)
Outcome	Discharged home stable (46.5%) Discharged to nursing facility (51.5%) Deceased (1.2%)

**Figure 2:**
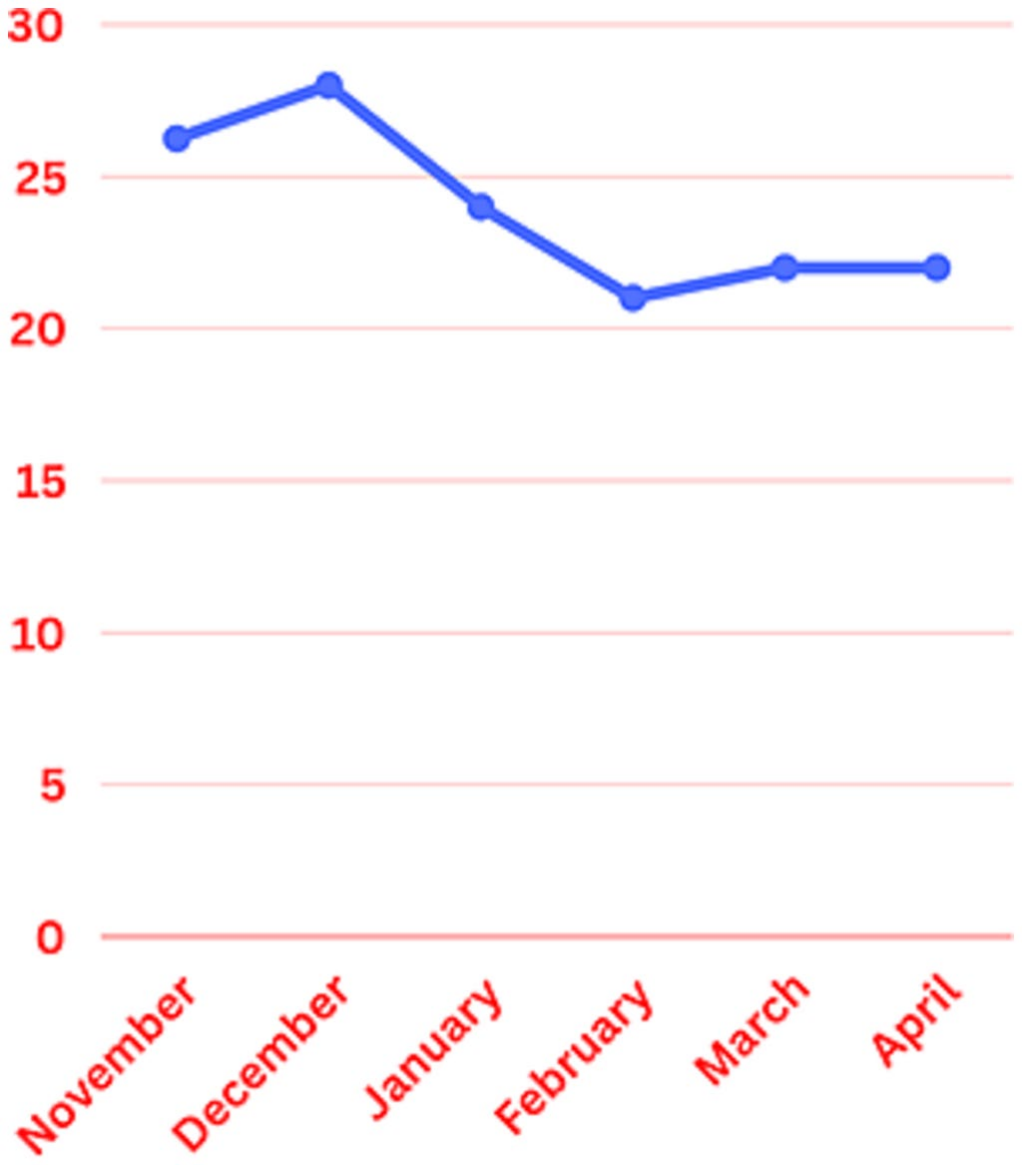
Mean monthly duration of EEG monitoring.

Mean monthly duration of the EEG monitoring was calculated across January through April. It was 24, 21.7, 22.6, and 22.1 h, respectively, (Figure 2). The sociodemographic and clinical profile of the patient population in the post implementation group is described in Table 2. Most common indications for EEG monitoring were seizures (22.8%) and altered mental status (27.7%). Most of the patients were on Internal Medicine floor services (40.7%) and up to 20% of the patients were under Neurology as primary service. Medical intensive care and neurocritical care services had 6 and 4% of the patient sample, respectively.

Out of 101 patients included in our study, 16 had a 2HELPS2B score of 0, while 47 and 38 had a score of 1 and 2, respectively. Mean duration of EEG monitoring for each subgroup was calculated. The subgroup with a score of 0 was 16.4 h, subgroups with scores of 1 and 2 had a mean duration of 22 and 26 h, respectively (Table 3).

**Table 3:** Mean duration of cEEG monitoring across the three low risk subgroups during preintervention and post intervention.

2HELPS2B score subgroup	Preintervention group	Postintervention group	Recommended duration of monitoring
2HELPS2B score 0	22.4 h (*n* = 14)	16.4 h (*n* = 16)	1 h
2HELPS2B score 1	27.6 h (*n* = 22)	22 h (*n* = 47)	12 h
2HELSP2B score 2	30.8 h (*n* = 9)	26 h (*n* = 38)	24 h

The mean monthly duration of EEG monitoring for patients was reduced from 28 h in December to 24, 21.7, 22.6, and 22.1 h in January, February, March and April, respectively, (Figure 2). There was about 22.5 percent reduction in the total duration of the EEG monitoring in the study group. The duration of cEEG monitoring for low-risk post intervention group ranged from 1 to 118 h. Twenty-four out of 101 patients included in the study had a cEEG duration above 24 h. Twelve out of those 24 patients were from January, 8 from February, 4 from March and none from April. Patients in the study group had a 2HELPS2B score of </=2, which falls under low-risk group. None of them had seizures during the EEG monitoring. A retrospective chart review was performed, and no clinical seizures were reported in these patients during the rest of the hospitalization, after the cEEG discontinuation.

Eighteen out of 19 patients were excluded from the analysis based on the presence of electrographic seizures during the EEG monitoring. One patient was excluded based on the age criteria. All 18 patients had an initial one-hour 2HELPS2B score of >2, with a mean score of five. These patients were in the high-risk group based on the predefined 2HELPS2B criteria. The mean age of this group was 62.4 years, slightly older than the patients included in the analysis. Seven patients were diagnosed with status epilepticus; five patients had intracranial hemorrhage and the rest had altered mental status as the indication for cEEG. Neurocritical Care Unit (NCCU) was the primary service for all 18 patients. Eleven patients died during the hospital stay, four patients were discharged to a nursing facility and three were discharged home in a stable condition.

## Discussion

This study was conducted in a three-site tertiary care hospital system as a part of the resident and fellow quality improvement project. cEEG is a valuable tool for seizure detection in the inpatient setting. There is limited guidance on the duration of EEG monitoring across various inpatient settings. UTMB has three campuses serving two community hospitals and one University hospital. Our institution is a level four epilepsy center with five full time EEG technicians. The University hospital campus has seven EEG machines, while the two community hospitals have three and two machines, respectively. The neurological critical care unit is located on the University hospital campus. Most of the providers in the community hospitals are non-epileptologists and medical intensivists. EEG monitoring is ordered for patients in intensive care and floor settings. Due to the diversity of providers and differences in the level of training in interpreting EEG reports, the duration of EEG monitoring even for lower risk patients can be highly variable and at times results in inadvertent prolonged EEG monitoring with no meaningful impact on medical management. In our workflow, the 2HELPS2B scoring system served as a practical tool to improve communication between the EEG reading team and treating providers. By incorporating an objective, validated risk score directly into the EEG reports, it allowed for clearer guidance on both seizure risk and the recommended duration of monitoring. This approach helped standardize communication across different provider groups and made it easier to interpret for those with limited EEG training.

During the preintervention period, a 2HELPSB score was retrospectively calculated. Low risk patients with a score of </=2 were further subdivided into three groups with scores of 0,1 and 2, respectively. All three groups have received a longer than recommended duration of EEG monitoring, with the highest discrepancy noted in the group with scores of 0 and 1. There was a reduction in the mean duration of EEG monitoring in the post intervention group across all three subgroups, although the monitoring continued to be longer than the recommended duration for the respective scores on 2HELPS2B scale.

None of the patients included in the study sample had a 2HELPS2B score of >2. Eighteen out of 19 patients that were excluded from the analysis due to having a seizure during the EEG monitoring had an initial 2HELPS2B score of >2 with a mean score of 5. Most of them were managed in neurocritical care setting and had a mortality rate of 16%, which was higher than the low-risk group with only 3%. These findings further support the validity of this tool in determining seizure risk and future occurrence of seizures based on a higher 2HELPS2B score and vice versa.

Although our aim was to reduce the mean cEEG duration by 30%, we did achieve a reduction of 22.5 percent. Upon further analysis, we do see a reduction of approximately 580 h of cEEG duration over a period of 4 months, with an average of roughly 6 h per patient. This is significant in terms of reducing the hours of expensive yet unnecessary EEG monitoring and also will also increase the access to the EEG resource. Our study did not directly look at the cost benefit analysis. Based on the study done by Abend et al. (12), reducing the cEEG duration from 48 to 24 h in low-risk patients saved the incremental cost of about 22,600 dollars in their study group.

One important observation made during our study was that most of the low-risk patients who had longer than 24 h of cEEG duration were from Intensive care unit and carried a diagnosis of cardiac arrest and anoxic brain injury. It is possible that the indication of EEG in such patients is neuro-prognostication. In patients who are heavily sedated and have received loading doses of antiepileptic medications, the treating team tends to continue EEG monitoring longer than the recommended cEEG duration based on 2HELPS2B criteria. These observations highlight the important consideration that the recommended duration of EEG monitoring should be adjusted according to the clinical indication and the patient’s sedation status.

## Limitations

Our study is limited by its small sample and short duration, which precluded subgroup analysis of 2HELPS2B scores of 0,1, and 2. Our study did not calculate the direct costs associated with prolonged EEG monitoring and did not address changes in the number EEG studies performed with the reduction the total duration of EEG monitoring in low-risk group. We used reduction in the average duration EEG monitoring as a surrogate marker of EEG related costs in our study. Our study is also limited in analyzing the effect of optimizing the EEG duration on patient outcomes. Although low risk patients in the postintervention group did not have any clinical seizures during the rest of the hospitalization after the EEG monitoring was discontinued, it is not entirely possible to rule out an electrographic seizure in these patients. The effect of sedation and loading dose of antiepileptic medications can affect the findings of EEG and it is uncertain if 2HELPS2B score can be applied with the same degree of validity in such situations.

## Conclusion and future directions

Despite limitations, 2HELPS2B scoring system implementation in cEEG reports institutionally can potentially optimize the duration of cEEG in low-risk individuals without compromising the seizure detection. By safely reducing the duration of cEEG in low-risk patients, we were also able to substantially reduce the cEEG hours over the period of study. This can not only reduce healthcare costs but improve access to EEG for patients who need it. Future studies should focus on understanding and implementation of 2HELSPS2B score in patients under heavy sedation.

## Data Availability

The raw data supporting the conclusions of this article will be made available by the authors, without undue reservation.

## References

[1] Herman ST Abend NS Bleck TP Chapman KE Drislane FW Emerson RG . Consensus statement on continuous EEG in critically ill adults and children, part I: indications. J Clin Neurophysiol. (2015) 32:87–95. doi: 10.1097/WNP.0000000000000166, PMID: 25626778 PMC4435533

[2] Hill CE Blank LJ Thibault D Davis KA Dahodwala ND Litt B . Continuous EEG is associated with favorable hospitalization outcomes for critically ill patients. Neurology. (2019) 92:e9–e18. doi: 10.1212/WNL.0000000000006689, PMID: 30504428 PMC6336162

[3] Ney JP Nuwer MR Hirsch LJ Burdelle M Trice K Parvizi J. The cost of after-hour electroencephalography. Neurol Clin Pract. (2024) 14:e200264. doi: 10.1212/CPJ.0000000000200264, PMID: 38585440 PMC10997216

[4] U.S. Department of Health and Human Services, Centers for Medicare & Medicaid Services (2014). Medicare provider utilization and payment data: physician and other supplier services. Available online at: https://www.cms.gov/medicare/medicare-fee-for-service-payment/physician-fee-schedule

[5] Shafi MM Westover MB Cole AJ Kilbride RD Hoch DB Cash SS. Absence of early epileptiform abnormalities predicts lack of seizures on continuous EEG. Neurology. (2012) 79:1796–801. doi: 10.1212/WNL.0b013e3182703fbc, PMID: 23054233 PMC3475619

[6] Westover MB Shafi MM Bianchi MT Moura LM O'Rourke D Rosenthal ES . The probability of seizures during EEG monitoring in critically ill adults. Clin Neurophysiol. (2015) 126:463–71. doi: 10.1016/j.clinph.2014.05.037, PMID: 25082090 PMC4289643

[7] Claassen J Mayer SA Kowalski RG Emerson RG Hirsch LJ. Detection of electrographic seizures with continuous EEG monitoring in critically ill patients. Neurology. (2004) 62:1743–8. doi: 10.1212/01.WNL.0000125184.88797.62, PMID: 15159471

[8] Rodriguez RA Vlachy J Lee JW Gilmore EJ Ayer T Haider HA . Association of periodic and rhythmic electroencephalographic patterns with seizures in critically ill patients. JAMA Neurol. (2017) 74:181–8. doi: 10.1001/jamaneurol.2016.4990 27992625

[9] Struck AF Ustun B Rodriguez Ruiz A Lee JW LaRoche SM Hirsch LJ . Association of an electroencephalography-based risk score with seizure probability in hospitalized patients. JAMA Neurol. (2017) 74:1419–24. doi: 10.1001/jamaneurol.2017.2459, PMID: 29052706 PMC5822188

[10] Struck AF Tabaeizadeh M Schmitt SE Rodriguez-Ruiz A Swisher CB Subramaniam T . Assessment of the validity of the 2HELPS2B score for inpatient seizure risk prediction. JAMA Neurol. (2020) 77:500–7. doi: 10.1001/jamaneurol.2019.4656 31930362 PMC6990873

[11] Moffet EW Subramaniam T Hirsch LJ Gilmore EJ Lee JW Rodriguez-Ruiz A . Validation of the 2HELPS2B seizure risk score in acute brain injury patients. Neurocrit Care. (2020) 33:701–7. doi: 10.1007/s12028-020-00939-x, PMID: 32107733

[12] Abend NS Topjian AA Williams S. How much does it cost to identify a critically ill child experiencing electrographic seizures? J Clin Neurophysiol. (2015) 32:257–64. doi: 10.1097/WNP.0000000000000170, PMID: 25626776 PMC4452395

